# Using EHRs for Heart Failure Therapy Recommendation Using Multidimensional Patient Similarity Analytics

**Published:** 2015

**Authors:** Maryam Panahiazar, Vahid Taslimitehrani, Naveen L. Pereira, Jyotishman Pathak

**Affiliations:** * Center for the Science of Health Care Delivery, Mayo Clinic, Rochester, MN, USA; † Division of Cardiovascular Diseases, Mayo Clinic, Rochester, MN, USA

**Keywords:** patient similarity, electronic health records, heart failure

## Abstract

Electronic Health Records (EHRs) contain a wealth of information about an individual patient’s diagnosis, treatment and health outcomes. This information can be leveraged effectively to identify patients who are similar to each for disease diagnosis and prognosis. In recent years, several machine learning methods^1^ have been proposed to assessing patient similarity, although the techniques have primarily focused on the use of patient diagnoses data from EHRs for the learning task. In this study, we develop a multidimensional patient similarity assessment technique that leverages multiple types of information from the EHR and predicts a medication plan for each new patient based on prior knowledge and data from similar patients. In our algorithm, patients have been clustered into different groups using a hierarchical clustering approach and subsequently have been assigned a medication plan based on the similarity index to the overall patient population. We evaluated the performance of our approach on a cohort of heart failure patients (N=1386) identified from EHR data at Mayo Clinic and achieved an AUC of 0.74. Our results suggest that it is feasible to harness population-based information from EHRs for an individual patient-specific assessment.

## Introduction

In Precision Medicine, the ability to match the right drug with the right dose to the right patient at the right time is vital^2^. This could be facilitated with the comparison of a new patient with patients having similar characteristics such as co-morbidities and pharmacotherapies. In the recent past, several statistical and machine learning methods have been proposed^3,4^ for analyzing patient similarity. However, the focus has primarily been on applying diagnosis data from EHR for the learning task. In this work, we adopt a more holistic view, and consider different sources of information from EHR including lab results, medications, comorbidities and demographics to develop a multidimensional approach for assessing patient similarity using machine learning techniques. The similarity assessment has the potential to aid clinical decision-making and therapy recommendation at the point-of-care. In particular, we applied a multidimensional patient similarity technique to investigate response to therapy in patients diagnosed with Heart Failure (HF) using various characteristics In the following sections, we describe our methodology for multi-dimensional similarity assessment and present preliminary findings on a cohort of HF patients (N=1386) derived from EHR data at Mayo Clinic.

## 1. Methods

In this study, we consider following variables from the EHR data to study and categorize the patients:

- Lab results including Lymphocytes, Cholesterol, Sodium and Hemoglobin.
- Medications including Angiotensin Converting Enzyme (ACE) inhibitors, Angiotensin Receptor Blockers (ARBs), β-adrenoceptor antagonists, (β-blockers), Statins, and Calcium Channel Blockers (CCBs).
- Demographics including age, gender, ethnicity and race.
- 26 co-morbid conditions as defined by the Chronic Conditions Data Warehouse from the Center for Medicare and Medicaid Services.^5^
- Echocardiogram measurements including ejection fraction.
- Vital signs including blood pressure, and body mass index (BMI).

For identifying a cohort of HF patients, we applied four eligibility criteria: (1) A diagnosis of HF based on the ICD-9-CM code (428.x); (2) An ejection fraction (EF) measurement less than 50% within one month of the HF diagnosis; (3) Another EF measurement between 6 months and 15 month after the first EF measurement; and (4) No prior diagnosis of coronary artery disease, myocarditis, cardiomyopathy and aortic or mitral stenosis. From a cohort of 119,749 Mayo Clinic patients between 1993 and 2013, we identified 7827 patients with a diagnosis of HF (criteria 1). After applying criteria 2–4 and excluding 673 patients due to incomplete data, our final study cohort had N=1386 patients with HF. Table 1 represents the characteristics of the study cohort. We developed the following criteria to define response to HF therapy: Patients are under the “poor” response to therapy cateogory if the individual has less than 10% increase in their EF measurement(s) within 12 months after HF treatment initiation. Patients are under the “good” response to therapy category if the individual has at least 10% increase in their EF measurement(s) within 12 months after HF treatment initiation.

We designed two different approaches to measure the patient similarity and prediction of an appropriate treatment plan. The main difference between these two approaches is the way we cluster the patients. In the first approach, we cluster patients using two standard clustering algorithms (K-means and hierarchical clustering)^6^ and in the second approach, patients are clustered using a supervised technique based on the medication plan. We summarize these approaches as follows: (1) Split patients based on their response to medication (Good vs. Poor response categories as defined above). (2) Cluster patients that responded to medication using two different techniques: unsupervised and supervised (a) Unsupervised clustering including k-means and hierarchical clustering and (b) Supervised clustering using the medication plan as class variable. (c) Assign a label to each cluster, if the clustering is done by the unsupervised technique. We identified the label of each cluster based on the most frequent medication plan. (d) Measure the similarity of the new patient with each cluster created in the previous steps in order to determine the medication plan of each new patient. To measure the similarity of a new patient with each cluster, we propose a generalized Mahalanobis distance^7^. (e) Rank the similarities and choose the most similar cluster to the new patient. And finally, (f) Recommend the medication plan of the most similar cluster to the new patient.

We use *X_i_* = [*x_i_*_,1_, …, *x_i_*_,_*_n_*] ∈ ℝ*^i^*^×^*^d^* to represent the feature vector of patient *i*, where *i* = 1, …, *n* and *n* is the number of patients and *d* is the number of features. *y_i_* is the label assigned to the patient *i* with *y_i_* ∈ {1,2, …, *L*} and *L* is number of class labels and in our case, the number of medication plans. Medication plan is based on using drug or combination of drugs with specific dosages during the treatment. The generalized Mahalanobis distance between patient *X_i_* and cluster *C* with means *μ* = {*μ*_1_, …, *μ_n_*} are defined as follows

$$d_{S}(X_{i},C)=\sqrt{{\left(X_{i}-\mu \right)}^{T}S(X_{i}-\mu )}$$

where *S* ∈ ℝ*^d^*^×^*^d^* is a Symmetric Positive Semi-Definite (SPSD) matrix. We use the Mahalanobis distance to measure the similarity between a patient and a cluster of patients to find out which cluster is the most similar ones to selected patient.

## 2. Results

**Figure 1:**
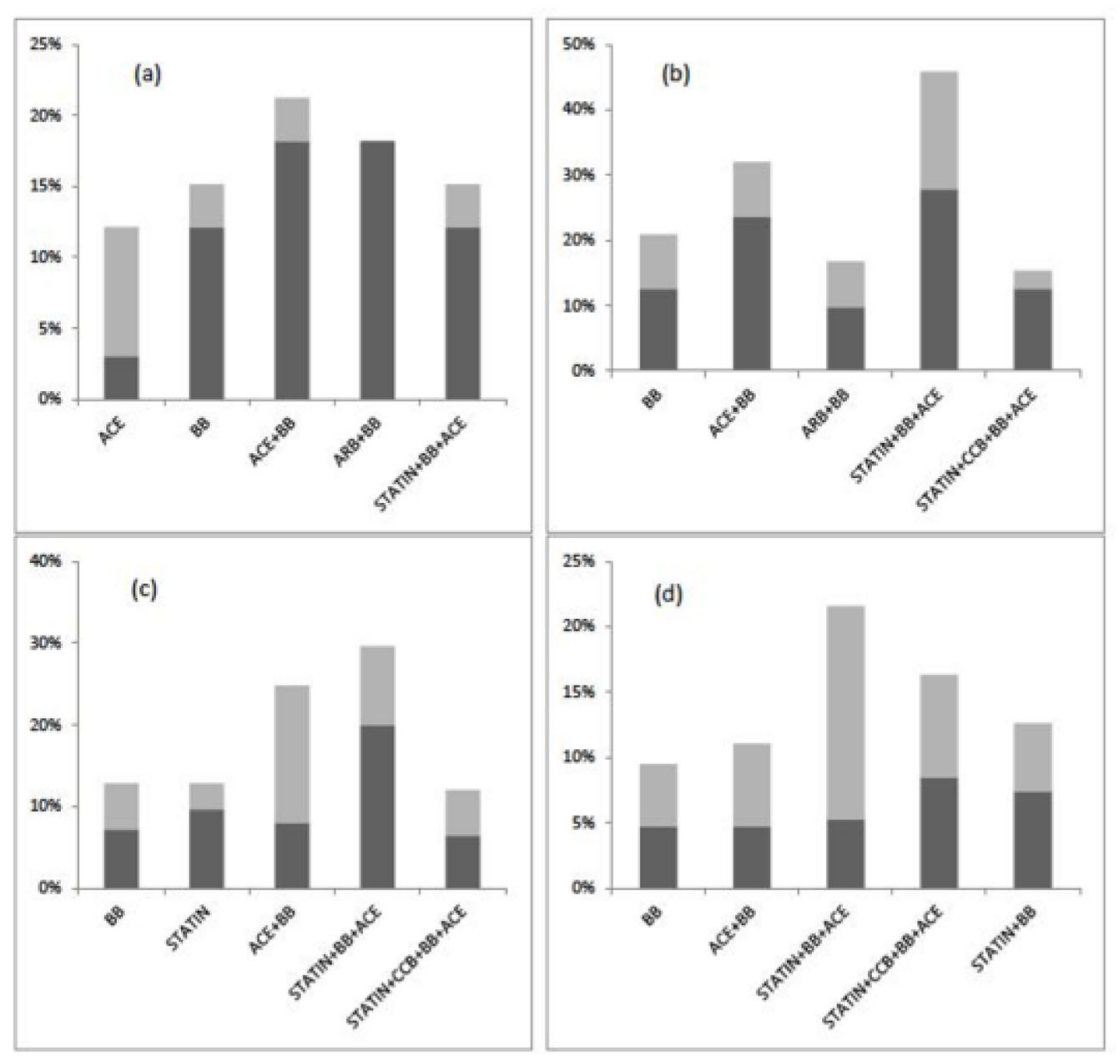
a) Medication Plans for Patients with EF<10%, b) Medication Plans for Patients with 10% <= EF <20%, c) Medication Plans for Patients with 30%<=EF<40%, d) Medication Plans for Patients with 40% <= EF < 50%

Our objective in this study was to propose an approach to use patient similarity techniques in order to determine the medication plan for a new patient based on the EHR data. To this end, we defined a patient similarity framework, allowing us to exploit the similarity based medication recommendation. We calculated the distribution of medication plans in our cohort. 57% (N=790) of the patients responded to HF therapy and their EF measurements increased by at least 10% after six months from the first EF measurement and initiation of HF therapy. In our cohort, we detected 28 different medication plans as combination of 5 medication classes. The results show that the combination of ACEIs, BBs and Statins is the most popular medication plan in our cohort with 17% (N=241) of the patients being prescribed this combination therapy, and with more than 50% (N=118) demonstrating an improvement in EF by at least 10%. The next common plan is ACEIs and BBs. More than 12% (N=166) of the patients were prescribed ACEIs and BBs and 51% (N=85) of the patients demonstrated good response to therapy. Note that statins and BBs are commonly prescribed to HF patients, which affirm the clinical practice guidelines. Figure 1 represents the frequency of medication plans across different EF intervals. Each figure shows the first 5 frequent medication plans for specific EF values less than 50%.

We calculated the AUC values for validating three different clustering approaches (supervised clustering=0.74, hierarchical clustering=0.71 and k-means clustering=0.69). To obtain robust area under curve (ROC) and avoid any chance of over fitting, we performed 10 fold cross validation in each run such that 70% (N=970) of the cohort was used to cluster patients and the remaining 30% (N=416) for testing and determining the medication plan. Then, we considered different cut points starting from 50% and finally calculated the average for each fold.

Regarding the validation process, it is noticeable that the training patients are clustered using different methods and then a medication plan is assigned to each test patient based on the similarity assessment. For unsupervised clustering, the number of clusters (N=7) in both k-means and hierarchical clustering is determined by cross validation analysis. Whereas for supervised clustering, due to a larger number of clusters (N=28) we applied agglomerative clustering to merge the smaller clusters with the bigger ones. If the number of patients under a specific medication plan or cluster is less than 5% of the whole population, we called it small cluster and merged it with one of the large clusters (more than 5% patients).

Our criterion to find a match to merge for each small cluster is similarity between the elements of each plan. For example, {Statins, CCB, ACE} as a small cluster is merged with {Statins, CCB, ACE, BB}. Although it is possible that small clusters are related to patients with very particular characteristics, we did not investigate those aspects in this study.

Table 2 represents the performance of different approaches tried in our analysis. The results suggest that high specificity of different approaches leads to the creation of that clusters are highly separated from each other and highly dense within each other.. Further, since in the first clustering approach, the patients are clustered using a supervised approach, there is no error in the clustering part and the whole error is related to the merging clusters and measuring the similarity between the patient and cluster.

## 3. Discussion

We applied patient EHRs for inferring an individual patient’s response to HF therapy. For this task, we use patient-specific information from the EHR, including medical co- morbidities, laboratory measurements, ejection fraction, vital status and demographics to identify similar patients, and subsequently predict HF therapy response. Even though our preliminary results are promising, they require further validation in a larger cohort, potentially, across multiple different EHR systems.

## Figures and Tables

**Table 1:** Patient Characteristics for Heart Failure Study Cohort (N=1386 unique patients)

Characteristics	Value	Characteristics	Value	Characteristics	Value
Age (years)	77±13	Myocardial infarction	28.1%	Depression	22%
Sex (male)	65	Acquired hypothyroidism	15.9%	Glaucoma	8.6%
Race (White)	96%	Alzheimer	49.9%	Hypertension	82.8%
Ethnicity	90	Atrial fibrillation	50.8%	Hyperlipidemia	78.9%
BMI	28.4±10.8	Anemia		Ischemic heart	71.2%
Ejection Fraction (EF) %	37±9.8	Benign prostatic	10.3%	Osteoporosis	12.7%
Hemoglobin g/dL	13±1.9	Breast Cancer	1.2%	Prostate cancer	6%
Sodium mEq/L	140±6.9	Chronic Kidney Disease	53.2%	Pulmonary disease	24.9%
Cholesterol mg/dL	155±42	Cataract	28.2%	Rheumatoid Arthritis	38.6%
Lymphocytes ×10(9)/L	1.53±0.78	Colorectal Cancer	0.9%	Stroke	11.4%
Asthma	9%	Diabetes	40.6%	Sys Blood Pres.	121±23

**Table 2:** Performance of Different Approaches

Method	Specificity	Sensitivity	F1	Accuracy	AUC
Supervised	0.85	0.52	0.58	0.77	0.74
Hierarchical	0.79	0.5	0.56	0.73	0.71
K-means	0.74	0.49	0.54	0.71	0.69
